# *In vitro* γ-ray-induced inflammatory response is dominated by culturing conditions rather than radiation exposures

**DOI:** 10.1038/srep09343

**Published:** 2015-03-20

**Authors:** G. Babini, J. Morini, G. Baiocco, L. Mariotti, A. Ottolenghi

**Affiliations:** 1 Department of Physics, University of Pavia, Pavia, Italy; 2 INFN, National Institute of Nuclear Physics, Sezione di Pavia, Pavia, Italy; 3 Department of Molecular Medicine, Biology and Medical Genetics Unit, University of Pavia, Pavia, Italy

## Abstract

The inflammatory pathway has a pivotal role in regulating the fate and functions of cells after a wide range of stimuli, including ionizing radiation. However, the molecular mechanisms governing such responses have not been completely elucidated yet. In particular, the complex activation dynamics of the Nuclear transcription Factor kB (NF-kB), the key molecule governing the inflammatory pathway, still lacks a complete characterization. In this work we focused on the activation dynamics of the NF-kB (subunit p65) pathway following different stimuli. Quantitative measurements of NF-kB were performed and results interpreted within a systems theory approach, based on the negative feedback loop feature of this pathway. Time-series data of nuclear NF-kB concentration showed no evidence of γ-ray induced activation of the pathway for doses up to 5Gy but highlighted important transient effects of common environmental stress (e.g. CO_2_, temperature) and laboratory procedures, e.g. replacing the culture medium, which dominate the *in vitro* inflammatory response.

The inflammatory response, with the *Nuclear transcription Factor kB* (NF-kB) as key player, has a fundamental role in managing the different functions of cells through the complex regulation of pro-survival pathways and both pro- and anti- inflammatory signals. The role of the inflammatory pathway is even more important at a systemic level: NF-kB is characterized by an ubiquitous presence in all cell types and with different functions, and its activation might be either protective for the organism, orchestrating the immune response against exogenous agents, or harmful, leading to cancer and other diseases if chronically altered or mutated^1^.

NF-kB was initially discovered more than 25 years ago^2^, but the variety of its functions still raises questions about how a limited set of mediators can be able to integrate such diverse stimuli (e.g. signalling proteins, environmental stresses, ionizing radiation, etc) to achieve cell type– and stimulus-specific responses^3^,^4^,^5^.

One of the reasons why the inflammatory response is far from being completely understood resides in its non-linear circuitry connections, based on negative/positive feedback loops. NF-kB is kept inactive by one of the isoforms of its *Inhibitor protein kB* (IkB)^6^,^7^ which binds to NF-kB sequestering it to the cytoplasmic compartment. When a stimulus triggers the pathways the *IkB kinase* (IKK) phosphorylates the complex NF-kB-IkB, causing the degradation of IkB and allowing NF-kB molecules to translocate to the nucleus and to bind to kB motifs present in the promoters of several genes. Therefore, NF-kB has the important function of regulating the transcription of large numbers of genes (e.g. pro-survival, pro-inflammatory, etc) including its own inhibitor, thus causing a negative feedback loop governing this pathway. A simplified scheme of NF-kB circuitry is shown in Fig. 1.

Several signalling proteins, such as pro-inflammatory cytokines (e.g. TNF-α, IL-1β, IL-6, etc^7^,^8^,^9^,^10^) and lipopolysaccharide LPS^8^,^11^,^12^, have been widely used to test the non-linear responses of the NF-kB pathway, varying the intensities of the stimulus, comparing prolonged *versus* transient exposures and comparing the effects of different exogenous agents, including ionizing radiation^9^,^13^,^14^,^15^.

NF-kB is also known to play an important role in the cellular responses to radiation injury. Ionizing radiations are well known for causing DNA damage and inducing the formation of Reactive Oxygen Species (ROS), which in turn can activate several inside-out and outside-in cell signalling cascades^16^,^17^,^18^,^19^,^20^,^21^. Several groups already investigated, with *in vitro* systems, the effects of different doses and qualities of ionizing radiation on the NF-kB regulatory dynamics^22^,^23^, showing a clear perturbation to the overall system response at high doses of X-rays or high LET ions, particularly at more than 10 Gy. Such results represent a good starting points to shed light on the mechanisms and temporal dynamics underpinning NF-kB signalling, especially when moving to moderate and low doses (less than 5 Gy), which might be of interest for mimicking healthy tissue exposures during radiotherapy, where effects in *in vivo* models have been reported^14^,^24^.

The well-known importance of NF-kB activation as critical regulator of inflammation and in innate/adaptive immunity led several research groups to address the possible correlation between NF-kB activity and radiation-induced genomic instability^25^ or bystander effects^20^,^26^,^27^,^28^, thus highlighting the central role of NF-kB also in non-DNA targeted effects.

Subsequent reports involving the use of non-ionizing radiation (e.g. UV light), with the purpose of observing possible synergic effects in the system, showed that an induced chronic inflammation followed by an UV-exposure can cause a translational inhibition at the level of the endoplasmic reticulum (ER)^29^. Further studies also highlighted the correlation between the NF-kB activation and post-translational modifications^30^ and with the activation of the Unfolded Protein Response (UPR)^31^,^32^,^33^.

In this scenario the role played by common environmental stresses has also to be carefully considered. Indeed, *in vitro* experiments have demonstrated that stresses due to pH variations (CO_2_ and temperature variations being strongly connected to this phenomenon^34^), addition of growth factors^35^ and other stimuli (e.g. levels of ROS^36^) are able to perturb the NF-kB signalling pathway.

The purpose of this work is to characterize the dynamics and the perturbation of the NF-kB activation in AG01522 cell fibroblasts due to different stressors. To this aim, we performed time-series analysis of the nuclear NF-kB component. The NF-kB pathway was tested under different combinations of treatments: the responses to different doses of γ-rays and to the addition of lipopolysaccharides (LPS) were investigated themselves and combined with the effect of laboratory procedures. We can conclude that both medium change and temperature/CO_2_ variations are able to induce a strong perturbation at the level of the NF-kB pathway response, while no radiation effects are measurable for γ-rays exposures up to doses of 5 Gy in our experimental setup.

## Methods

### Cell Culture

Normal Human skin Fibroblasts (AG01522) were used for our experiments. Cells were obtained from Coriell Cell Repositories (Camden, NJ, USA). The cell line was maintained in Eagle's medium alpha (α-MEM, Biowest, France) with 10% Foetal Bovine Serum (FBS), supplemented with 100 IU/ml penicillin and 100 ng/ml streptomycin. Cells were seeded in T75 flasks in 10 ml culture medium, incubated at 37°C in humidified atmosphere with 5% CO_2_. Cultures were fed with fresh α-MEM every three/four days. Cells were cultured until 80–90% confluent monolayers were obtained before being sub-cultured using 0.25% Trypsin-EDTA solution. One week prior to the experiment, cells were seeded at an initial density of 2–3 × 10^5^ cells in a T75 flask and the medium changed after three days, without further handling the samples prior to the experiment. Unless otherwise specified, all cell culture reagents were purchased from Sigma-Aldrich Corporation (St Louis, MO).

### Irradiation procedure and collection of samples

Irradiations were performed with the CGR Alcyon II facility at the Policlinico San Matteo hospital, in Pavia^16^. For the irradiation of the samples, the ^60^Co γ-ray source was situated under the treatment plane and the samples were irradiated from the bottom at a dose rate of 0.5 Gy/min. A 5 mm layer of plexiglass was placed under the flasks to take account of γ-ray dose build-up.

Unless otherwise stated, cells were irradiated 45 minutes after the medium change with a dose in the range from 25cGy to 5 Gy. Control “sham” samples (0 Gy) were not irradiated but treated exactly like irradiated samples (e.g. time outside incubator, transfer to the irradiation facility, etc). After the irradiation, the samples were put back in the incubator. At subsequent time points conditioned culture media were collected and cells were either trypsinized for nuclear/cytoplasmic extraction or fixed for immunofluorescent studies.

### LPS treatment protocol

AG01522 were stimulated through the addition of different concentrations of lipopolysaccharide LPS (Cat. n°L4524, Sigma-Aldrich corporation, St. Louis, MO, USA) in order to induce NF-kB activation and an inflammatory response. The chosen concentrations were either control (0 ng/ml, PBS), 5 ng/ml or 50 ng/ml. Cells were then either harvested for nuclear/cytoplasm extraction or fixed for immunofluorescent studies.

### Protein total amount quantification (BCA Protein Assay)

Immediately after collection, cell pellets underwent a nuclear/cytoplasmic extraction protocol (Cayman Chemicals, Michigan, USA) and were stored at −20°C in order to be subsequently analyzed (within a few days after the collection).

The total proteins in the nuclear/cytoplasmic extracts were estimated by the BCA Assay (Abcam, Cambridge, UK). The assay was performed on 96-wells plates with samples properly diluted in order to have a final concentration within the range 0.5–1500 μg/ml and following the manufacturer's protocol.

### NF-kB Enzyme-Linked ImmunoSorbent Assay (ELISA Assay)

NF-kB nuclear concentrations were determined with 96-wells ELISA Assay kits, specifically coated for NF-kB-p65 subunit (Cayman Chemicals, Michigan, USA), performed on nuclear extracts. Transcription factor concentrations were measured with a microplate reader (DV990win6, GDV, Italy) at 450 nm wavelength. All standards, controls and samples were run in duplicate. The assays were performed as described by the manufacturer.

### Immunocytochemistry

Twenty four hours prior to the treatment, 5 × 10^4^ cells/ml were seeded on cover slips in a 6-well plate containing 2 ml of complete α-MEM.

At different time points after the treatment (LPS stimuli or medium change), cells were washed with Phosphate Buffered Saline (PBS) and fixed with ice-cold 70% ethanol for 2 hours at −20°C. In order to detect NF-kB proteins, cells were washed twice with PBS-0.05% Tween-20 and then permeabilized for 5 minutes at room temperature with 0.1% Triton X-100 in PBS. Cells were then incubated 1 hour at room temperature with PBS-1% BSA blocking agent. NF-kB primary antibody in PBS-0.2% Tween-20 (PBT) (Epitomics, #1546-1, CA, USA) was applied overnight at 4°C and anti-rabbit AlexaFluor-488 secondary antibody in 0.2% PBT (Molecular Probes) was applied for 45 min at room temperature. Finally the cover-glass was stained for 5 min with Hoechst 33342 for nuclear counterstaining prior to mounting the cover slips with MOWIOL 4-88 (Calbiochem, Cat# 475904, Darmstadt, Germany). For each slide, 10 images were captured with a CCD camera (RETIGA 2000R, QImaging, Canada) coupled with a fluorescent microscope (Olympus BX51, Olympus, Japan). Exposure times of 0.5 and 3.5 seconds were respectively used to collect localization images of nuclei and proteins. All images were collected using a 40× magnification objective (at least 50 cells were evaluated for each slide). Images were processed and analyzed with a semi-automatic ImageJ script based on the ImageJ built in plugin MRI-Cell Image Analyzer (Supplementary Material 2). The script allowed the operator to check each image background and immediately obtain the calculation of the ratio between nuclear and cytoplasmic NF-kB presence, defined as follows:
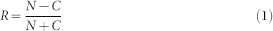
where N and C represent the integral fluorescence intensities of NF-kB in the nuclear and cytoplasmic compartments, respectively. As it can be easily seen, R values range between R = -1, in the case of fully cytoplasmic, and R = + 1, for fully nuclear NF-kB localization^37^.

### Theoretical model

**Figure 1:**
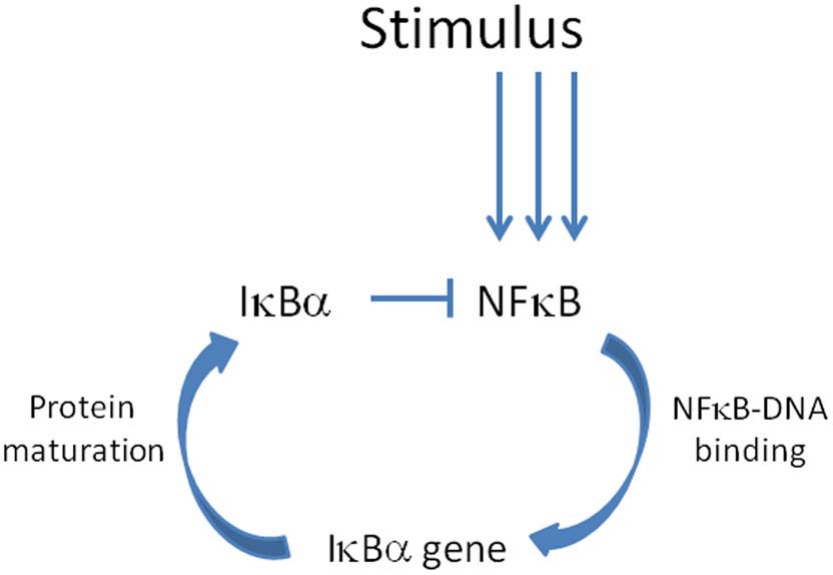
Simplified view of the NF-kB pathway.

The theoretical model was developed on the basis of a simplified scheme of the NF-kB circuitry (Fig. 1). The resulting analytical expression for the concentration of nuclear NF-kB (i.e. freed from its own inhibitor) as a function of time reproduces the temporal dynamics of the activation.

In particular, the model is based on the inhibiting function of IkB isoforms (e.g. IkBα), which also have high turnover rates (and therefore a short half-life time of approximately 10 minutes when the IkBα is alone). When an extracellular signal triggers the cell-surface receptors and subsequently activates the kinases IKK, the inhibited NF-kB is freed and can be translocated to the nucleus where it can bind to the kB motifs on the DNA and allow the transcription of a large number of genes, including its own inhibitor IkBα, which finally closes the negative feedback loop.

Due to the presence of several steps between NF-kB activation and its inhibition by newly created IkB, the whole system might be considered at first instance as a long ranging feedback process involving a long time delay, showing the important advantage of speeding up the system response to external changes^38^.

The NF-kB activation/inhibition can therefore be considered as driven by a negative feedback loop, mathematically represented by the general differential equation of a damped harmonic oscillator: 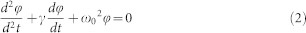
which, in the underdamped case, gives rise to the general solution *φ(t)*:
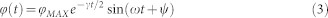
The amplitudes of the oscillations, often referred to as the *envelope*, are driven by the product of a maximum amplitude constant ***φ_MAX_*** and the exponential decay given by the damping coefficient ***γ***, while the sinusoidal component is described by the pulsatance ***ω*** and the phase ***ψ***.

Back to our system of interest, the particular solution which takes into account the final homeostatic level ***k_homeostasis_*** of nuclear NF-kB amount as a function of time can be written as:
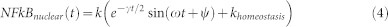
where **k** is a general normalization constant.

### Statistical analysis

If not explicitly stated, the reported values for each time point and condition are obtained as the mean of the results from 3 different biological samples, and the error bars represent their standard deviations.

Regarding the immunocytochemistry analysis, the R value is the result of the average of 10 different fields (40× magnification) and the total number of evaluated cells is always greater than 50. In this case, error bars represent the standard error of the mean (SEM) of the 10 fields.

The fit with the mathematical function given in Eq. (4) was obtained using MINUIT, a package developed at CERN and commonly used to find the best values of a set of parameters for a user defined fit function. The best fit function was obtained by minimizing a chi-square value taking into account both the y-axis error bar and a realistic uncertainty in the temporal scale, i.e. error bars of ±5 minutes on the x-axis. Free fit parameters(***γ***, ***ω***, ***ψ***, ***k_homeostasis_*** and ***k***), obtained from the chi-square minimization, are stored and subsequently used for data and curve plotting with the command-line program Gnuplot.

## Results

### NF-kB pathway is activated after Change of Media

In order to characterize our *in vitro* model we first evaluated the effect of a Change of culture Media (CM) at t = 0 h, through a NF-kB ELISA assay on nuclear extracts, with no additional treatments or radiation exposure. All sham (control) samples underwent the irradiation protocol as described in the previous section, being therefore subjected to the same environmental stress as the irradiated samples.

**Figure 2:**
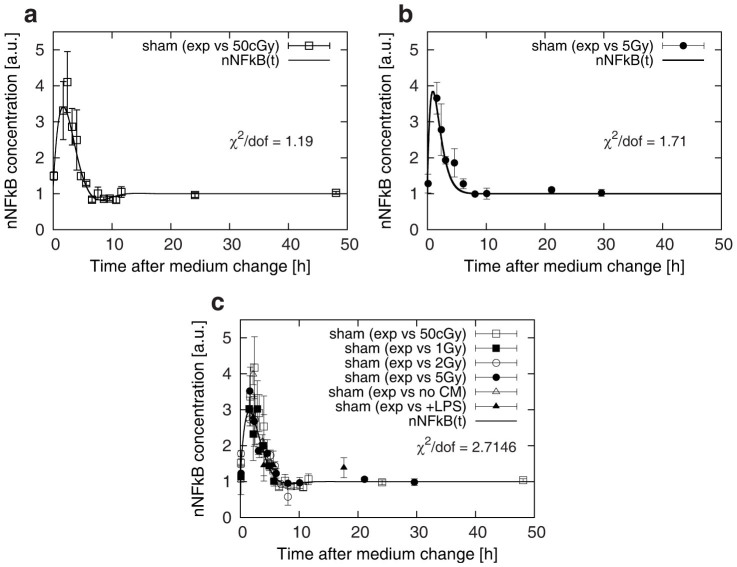
Temporal dynamics of nuclear NF-kB concentration in control samples following a change of culture media at t = 0 h. Time-series data for the NF-kB concentration (arbitrary units [a.u.]) from sham treated samples (0 Gy) from experiments with, respectively, 50 cGy (a) and 5 Gy (b) irradiated samples, with the best fit curves obtained with Eq. 4. In panel (c) all data from sham samples (0 Gy and no LPS pre-treatment) are shown together, with a common normalization and best fit curve obtained with Eq.4. Reduced chi-squared (“χ^2^/dof”, i.e. chi squared divided by the number of degrees of freedom) values for the fit are reported in each panel.

In Fig. 2a and Fig. 2b we report the sham datasets (from two experiments with 0.5 and 5 Gy, respectively), fitted through the model of Eq. 4. Both datasets show a clear activation of the inflammatory response through the nuclear translocation of the transcription factor NF-kB.

Concerning data reproduction, as is evident from the sham dataset of the 0.5 Gy experiment (Fig. 2a) an oscillation in the nuclear NF-kB amount is reproduced in the best fit curve, which tends to justify the choice of an underdamped model. However, no oscillation is required to best fit data for the 5 Gy experiment sham condition (Fig. 2b), even if such data correspond to identical experimental protocols.

The difference in the shapes for the two fit curves resulting from equivalent conditions might be ascribed to the biological variability of the setup at short times, affecting data collection. Such variability is clearly reflected in the error bars of experimental points, which are systematically larger at shorter times, when the system is more perturbed and far from recovering the original homeostasis. Even though the uncertainty on the temporal scale is also taken into account, no common trend from these different datasets under the same conditions is obtained.

In order to recover a more general trend in the NF-kB kinetics after CM we performed a single fit to all collected sham datasets from independent experiments (Fig. 2c). As a result of the combined fit and normalization procedure an oscillation is evident in the NF-kB kinetics, even if less pronounced than the one obtained from fitting the single sham dataset from the 0.5 Gy experiments. Conversely, the quality of the fit is worsened, as indicated by the higher reduced chi-square value reported in Fig. 2c.

As is evident from all datasets and corresponding fit curve (Fig. 2c), the collective response of the cell population shows a maximal concentration of nuclear NF-kB at about 1.5–2 hours after CM, with a value approximately 3-fold higher than the homeostatic level. Independently of the possible presence of an oscillation, the system is able to recover to the equilibrium state within 5–6 hours post CM, which supports the hypothesis of a complete recovery. As experimentally observed by other groups^4^,^10^,^12^,^39^,^40^, this property can be lost when the system is more strongly perturbed, and this may result in the manifestation of more oscillations over time.

### No effects of radiation were observed up to 5Gy after CM

**Figure 3:**
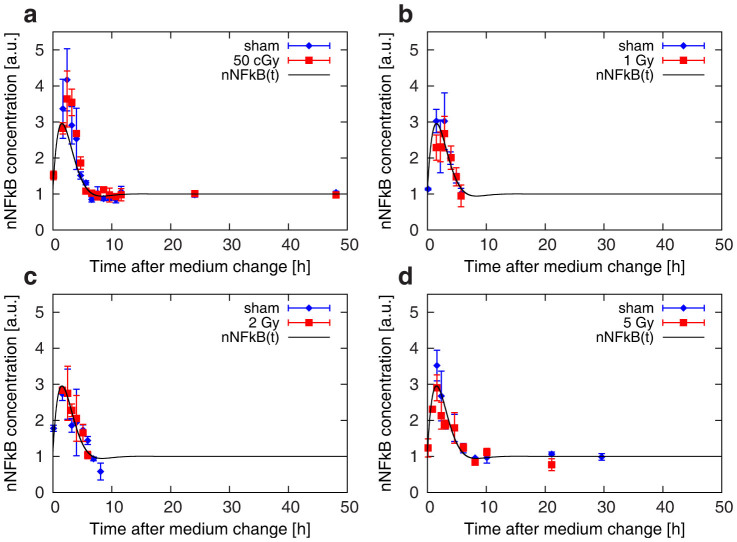
Temporal dynamics of nuclear NF-kB concentration in samples irradiated at different doses of γ-rays 45 minutes after the change of culture media at t = 0 h. Time-series data for the NF-kB concentration (arbitrary units) in samples exposed to 50 cGy (a), 1 (b), 2 (c) and 5 Gy (d) of γ-rays compared to the corresponding controls. Each experimental point is the average over three different replicas, and the error bar represents the standard deviation. In each panel, solid line is the best fit to all sham datasets, taken from Fig. 2c.

The nuclear amount of NF-kB following the exposure to different doses of γ-rays 45 minutes after the replacement of culture media was also measured. Data are shown in Fig. 3a–d, with panels corresponding respectively to radiation exposures with doses of 50 cGy, 1, 2 and 5 Gy.

As a guide for the eye in data reading, the best fit curve to all pooled sham datasets given in Fig. 2c is also plotted in each of the Fig. 3 panels. For each experiment, a peaked response at about 1.5–2 hours after CM is clearly observable, but differences in the dynamics appear negligible when comparing experimental data from irradiated samples and their corresponding controls.

### Environmental stress can perturb the NF-kB pathway

In order to show whether radiation effects are possibly masked by the stress induced by the CM, we performed two additional independent measurements of NF-kB activation without feeding the cells with fresh new medium prior to the radiation exposure, keeping all other steps of the protocol identical.

**Figure 4:**
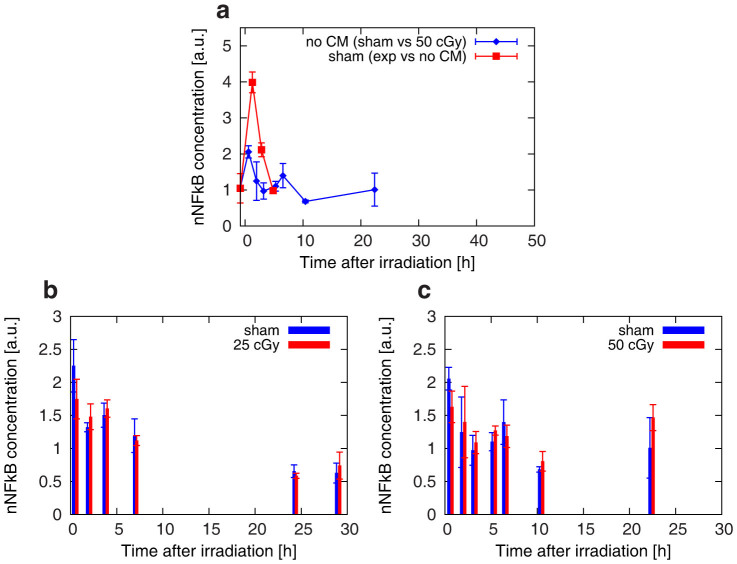
Temporal dynamics of nuclear NF-kB concentration in samples not affected by the replacement of culture media and exposed to low doses of γ-rays. (a) NF-kB concentrations (arbitrary units) for non-irradiated samples, with (red) and without (blue) a change of culture media at t = 0 h. Panels (b) and (c) show respectively data for the irradiations at 25 and 50 cGy and corresponding controls, without any change of media prior to the exposure. Each experimental point is the average over three different replicas, and the error bar represents the standard deviation.

A comparison between sham samples with and without medium change at t = 0 h (Fig. 4a) confirms that the perturbation to the NF-kB pathway is higher in the CM case, as it can be concluded from the differences observed between concentrations in the peak region at short time points.

When the culture medium is not replaced, the perturbation is induced by environmental stress only (pH/CO_2_/temperature variations due to transfer to the irradiation facility - see Supplementary Material 1 on the kinetics of cell culture media pH after CM).

Despite the observed difference, the system is able to recover within the same time interval, reaching the original homeostasis.

Having established a less stressed reference condition we turned to the investigation of radiation-induced perturbations of the NF-kB pathway on cells not affected by CM. In this case, we focused our attention on relatively low doses, where non-linear responses might have important effects.

As it is evident from the data comparison between samples irradiated at 25 and 50 cGy (Fig. 4b and Fig. 4c, respectively) and the corresponding controls, no clearly observable additional perturbation of the system is detected, even if the condition of cells at the exposure is less perturbed.

### Cells pre-treated with LPS confirm a major activation of NF-kB also after CM

As a positive control for our experimental setup, a comparison between LPS pre-treated cells (with 5 ng/ml for 4 h prior to the replacement of the medium) and cells affected only by CM was also carried out. Indeed, LPS is well-known to affect the overall inflammatory pathway response^8^,^11^,^36^ and represents a good test for activation dynamics of NF-kB in our *in vitro* model.

**Figure 5:**
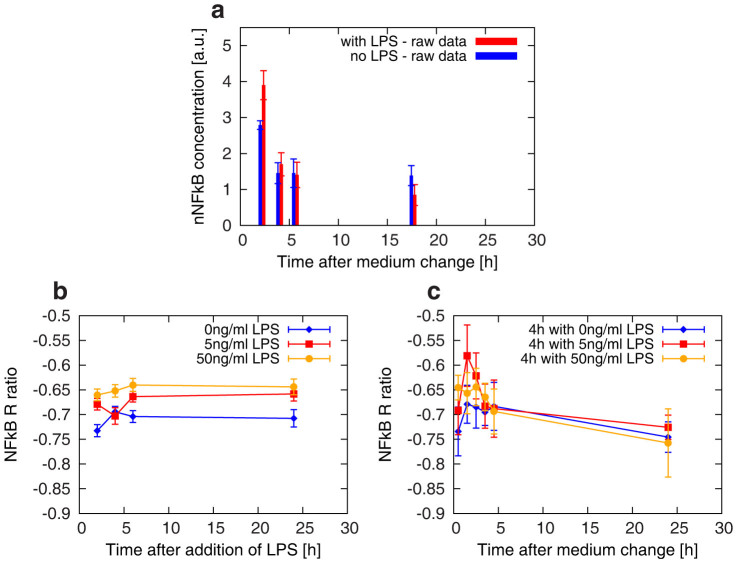
Effects of the LPS pre-treatment on nuclear NF-kB concentration and on NF-kB recruitment to the nuclear compartment following a change of culture media at t = 0 h. (a) NF-kB concentrations (arbitrary units) for samples subjected to a change of culture media at t = 0 h, with (red) and without (blue) a LPS pre-treatment (4 hours at 5 ng/ml). Each experimental point is the average over three different replicas, and the error bar represents the standard deviation. The ratio of nuclear versus cytoplasmic localization of NF-kB (R ratio of Eq. 1) is plotted against time in panels (b) and (c), respectively without and with a change of culture media occurring at t = 0 h, and for three different initial LPS concentrations during 4 hours of pre-treatment: control-PBS(blue), 5 ng/ml (red), 50 ng/ml (yellow). Error bars represent the Standar Error of the Mean from 10 different fields of fluorescent images. Lines are guide for the eye.

Time-series data on nuclear extracts with/without LPS pre-treatment are reported in Fig. 5a. In both cases, the change of culture media induces an increase in the amount of nuclear NF-kB, which later decreases to the same homeostatic level, suggesting the complete recovery of the cells from the LPS-induced inflammatory state.

The difference observed at the shortest time point between NF-kB concentration with/without pre-treatment suggests an initial higher difficulty for the cells to withstand both an active inflammatory state (caused by the LPS) and the subsequent stress of the change of medium.

To shed further light on the effect of LPS treatment and to gain insight into the underpinning mechanisms and dynamics, we turned to the investigation of cellular localization of the NF-kB molecules.

To this aim, immunocytochemistry-based studies have been performed in order to evaluate the localization of NF-kB within the cells and, in particular, to quantify the ratio between the nuclear and cytoplasmic components. Details are given in the Material and Methods section and Supplementary Material 2.

In Fig. 5b we report the value of the R ratio (as defined from Eq.1) as a function of time after adding different concentrations of LPS (0 ng/ml, 5 ng/ml or 50 ng/ml). The data show an increased nuclear NF-kB concentration with increasing concentration of LPS, also highlighting a prolonged perturbation of the system over time: the cell population shows an adaptation effect at 6 hours after treatment, when the inflammatory pathway has already reached different homeostatic levels in correspondence to the different treatments.

When replacing the culture media after LPS pre-treatment for 4 hours, the response of the system does not vary linearly with increasing LPS concentration (Fig. 5c). In fact, the maximal nuclear NF-kB amount for cells pre-treated with 5 ng/ml LPS is higher than both in the corresponding control samples and in cells pre-treated with 50 ng/ml of LPS, thus corresponding to a higher original inflammatory state. On the other hand, at late time points after medium replacement, differently pre-treated cells completely recover and reach the same final homeostatic level, showing no persistent effect of the different inflammatory states. This is consistent with the finding of a common homeostatic level for the NF-kB time dependent concentrations shown in Fig. 5a.

## Discussion

While it is well known that moderate doses of radiation are able to activate inflammation^14^,^24^, no clear effect on NF-kB activation induced by γ-rays exposures up to 5 Gy could be detected in our cellular system through time-series measurement with ELISA assays. The perturbation of the system observed in terms of a transient peaked activation could be attributed to the culture media replacement and to environmental stress (e.g. temperature, CO_2_) only. In the control case without medium replacement, the same temporal kinetics for NF-kB activation was observed, albeit with a reduced strength of the perturbation.

The transient activation induced by CM was finally found to be more pronounced if cells were previously treated with LPS at different concentrations, corresponding to a slight difference in nuclear/cytoplasmic NF-kB localizations over time, as revealed by additional immunocytochemistry analyses.

Considering the specificity of all investigated stimuli and the reproducible temporal kinetics, a major candidate for the observed effects is unspecific Endoplasmic Reticulum (ER) stress. The NF-kB activation level is based on a trade-off between the IkB rates of degradation, translation and complete maturation. The free IkB form has a half-life time of approximately 10 min^29^. This causes large oscillations in NF-kB activation, when the free pool of IkB is depleted due to a blocked protein formation, and eventually leads to a new inflammatory equilibrium if the original basal conditions are not restored. As seen also by other groups, LPS at different concentrations has important effects at the ER level, causing a not negligible stress and leading to reduced capabilities of new proteins translation and so-called Unfolded Protein Response (UPR) activation. The role of the ER in the NF-kB activation is therefore crucial and can also be very informative with respect to the radiation-induced NF-kB response.

Concluding, the results presented in this paper show that no radiation induced perturbation on the NF-kB pathway is measurable in our setup, up to moderate doses (5 Gy) of low LET radiation.

The same does not necessarily hold for high LET radiation exposure: different studies^22^,^30^ showed indeed an altered NF-kB activation at doses lower than the ones necessary to observe an effect with low LET radiation, i.e. 10/15 Gy. This might be ascribed to the impact of densely ionizing radiation on the integrity of organelles, which can result in the alteration of functions due to particle tracks traversing sub-cellular structures.

Conversely, we measured a complex and non linear response kinetics for the NF-kB pathway triggered by stresses due to common every day laboratory procedures, such as the change of culture media, or change in pH and temperature due to transport to irradiation facility. If the system undergoing such stresses is already in an active inflammatory state (due to the LPS pre-treatment in our case), the inflammatory response is even amplified. Biological interpretation of our data suggests unspecific ER stress as a major candidate for the observed effects.

A caveat is finally emerging from our study, namely the fact that results from *in vitro* research on inflammation may be influenced by common every day laboratory procedures, which affect the overall inflammatory response of the system, dominating the subtler effects induced by e.g. low to moderate doses of low LET radiation.

## Author Contributions

G.B., L.M. and A.O. designed the research; G.B., L.M. and J.M. performed the experiments; G.B. and G.B. analyzed and interpreted the data; G.B., G.B., J.M. wrote and edited the manuscript; A.O. critically read the manuscript; all authors reviewed the manuscript.

## Supplementary Material

Supplementary Information Supplementary material 1 & 2
