# Supplementary material for: In vitro γ-ray-induced inflammatory response is dominated by culturing conditions rather than radiation exposures
Source: Sci Rep. 2015 Mar 20;5:9343. doi: 10.1038/srep09343 (PMC4366819; doi:10.1038/srep09343)
Supplement: Supplementary Information — Supplementary material 1 & 2 [file srep09343-s1.pdf]

***In vitro* γ-ray-induced inflammatory response is dominated by culturing conditions rather than radiation exposures.**

G. Babini[1,2], J. Morini[1,2,3], G. Baiocco[1,2], L. Mariotti[1,2], A. Ottolenghi[1,2]*

[1] Department of Physics, University of Pavia, Pavia, Italy

[2] INFN, National Institute of Nuclear Physics, Sezione di Pavia, Pavia, Italy

[3] Department of Molecular Medicine, Biology and Medical Genetics Unit, University of Pavia, Pavia, Italy

* corresponding author email: gabriele.babini01@ateneopv.it

Supplementary material 1

Temporal dynamics of culture medium pH level

Supplementary data reported here concern the perturbation kinetics of pH in cell culture medium, with and without cells, and following environmental stresses only (time outside the incubator, transport to the irradiation facility) and/or radiation exposures.

Methods

One week prior to the experiment, cells were seeded at an initial density of 2-3x10^5 cells in a T75 flask in 10 ml of Eagle's medium alpha (α-MEM, Biowest, France) with 10% Foetal Bovine Serum (FBS), supplemented with 100 IU/ml penicillin and 100 ng/ml streptomycin. The medium was changed after three days, without further handling the samples prior to the experiment.

At different time points after the change of medium, the culture medium was collected from different T75 flasks (irradiated or not). A study of the pH level in cell conditioned culture medium samples was performed with the "BASIC 20" pH-Meter (CRISON Instruments, Spain. During both calibration and measurement the standard buffers and the samples were lightly stirred with identical

stirring speed to ensure the homogeneity of the samples. For a valid measurement of the pH, culture medium pH value was analyzed at a constant temperature (within ± 1°C).

Experimental data given in the following always represent the mean pH value of 3 biological samples, with error bars given as corresponding standard deviations.

Results

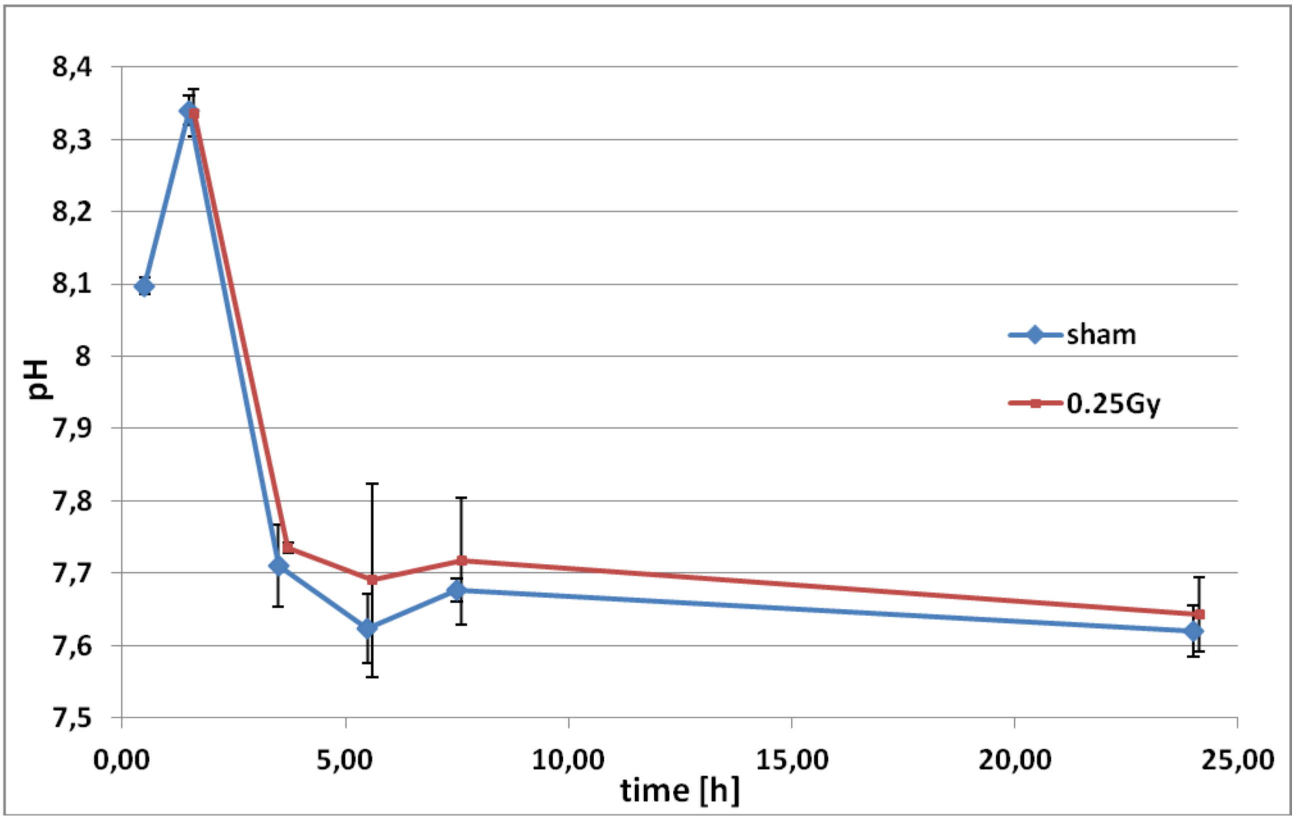

Figure S1.1 Comparison of the pH values measured in the culture medium collected at different time points for irradiated (0.25 Gy, red) and control (0 Gy, blue) samples. Lines are a guide to the eye.

Fig. S1.1 shows the comparison between pH values of culture media conditioned by irradiated cells (25 cGy) with the corresponding control. No effects of radiation can be observed, and a peak in the pH value is evident in both datasets at about 1.5 h, after putting the samples back in the incubator.

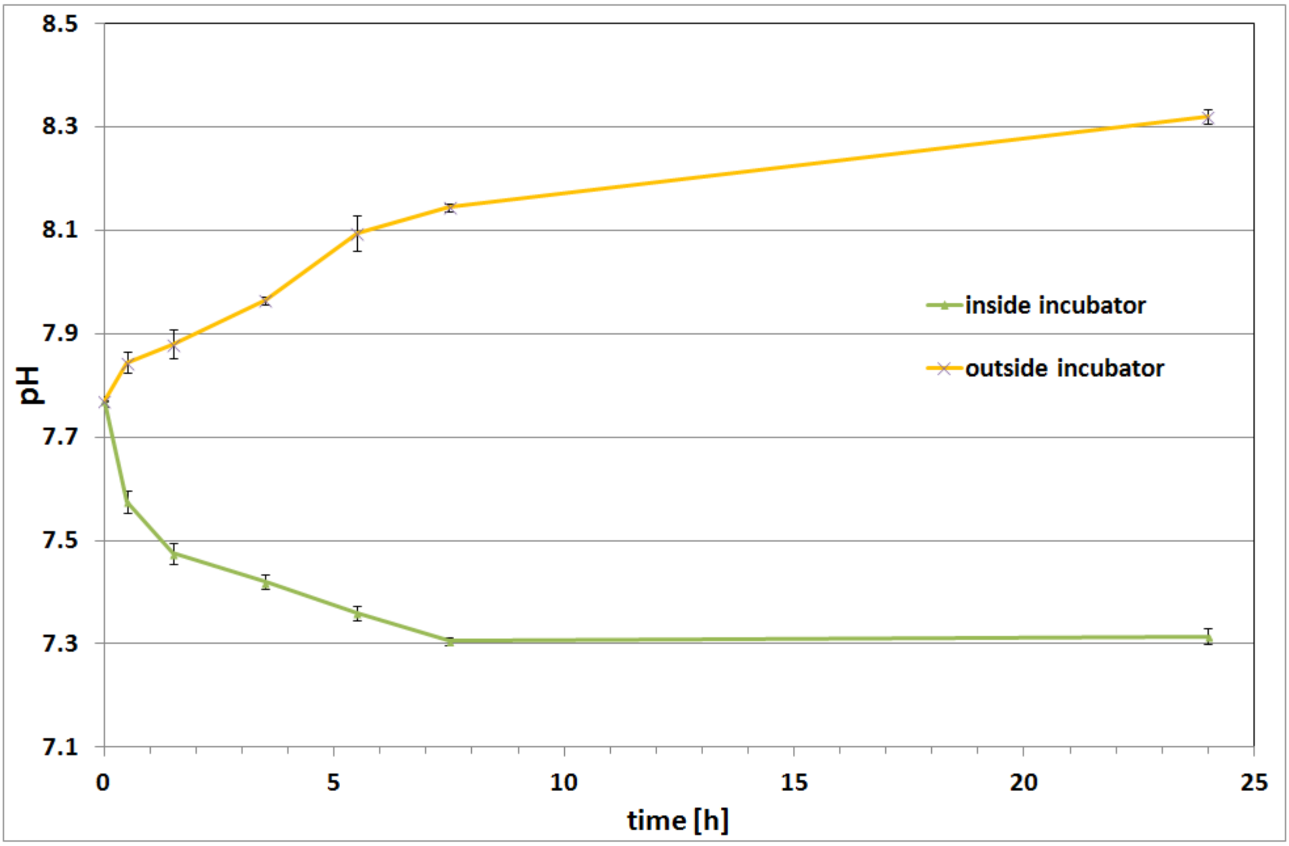

Figure S1.2 Comparison between the effect of the incubator environment (green) and room temperature conditions (yellow) on the pH of culture media samples alone (no cells), measured at different time points. Lines are a guide to the eye.

The measurement of pH values in culture media without cells was also performed, keeping the samples either in the incubator or outside (room temperature), in order to test the effect of environmental stress (temperature and CO_2 percentage).

Fig. S1.2 shows the difference in pH between media kept inside or outside the incubator: as expected, when left outside the incubator, CO_2 molecules leave the culture media causing an increase in the pH level.

Finally, we also measured the pH of media in flasks left for an hour at room temperature outside the incubator, and not transported to the irradiation facility. Results are reported in Fig. S1.3, and the dynamics of alkalinization (time outside the incubator) and subsequent acidification (flasks placed back in the incubator) are found to be compatible with results from the sham case, with observable but minor differences due to the different handling (differences in time outside the incubator, shaking due to transportation, etc.).

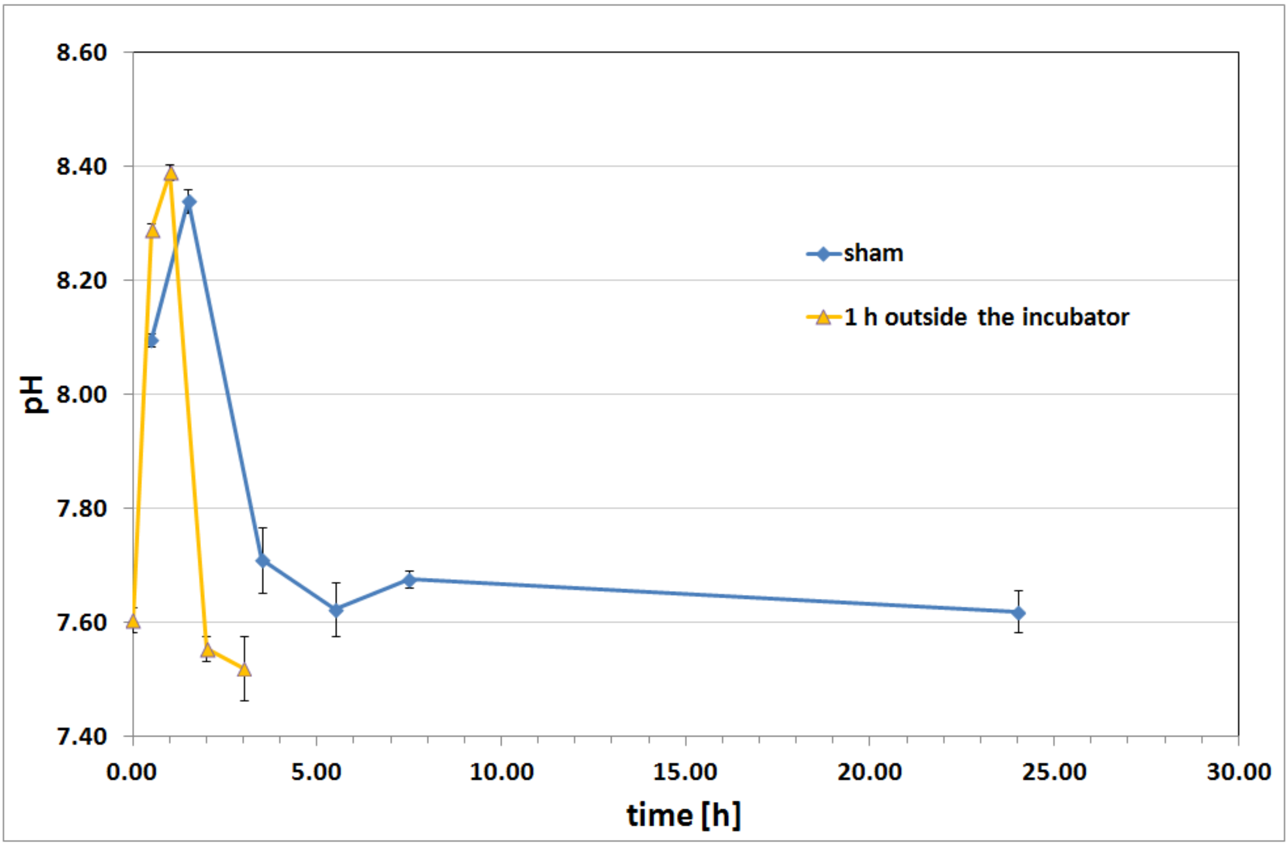

Figure S1.3 pH values measured in the culture medium for flasks left for 1h outside the incubator and then placed back in (yellow). "Sham" data are reported from Fig. 1 as a reference. Lines are a guide to the eye.

Supplementary material 2

We detail in the following the functioning of the macro developed to analyze fluorescence images for the immunocytochemistry analysis.

The starting point of the macro development has been the ImageJ built in plugin MRI-Cell Image Analyzer (Baecker & Travo, 2006). The idea of a homemade macro stems from the need of performing iterative image analysis: in the case of a large amount of images, the analysis is safer when made as automated as possible, always leaving the final control and check to the user, but reducing at most the numbers of operations/actions the user has to perform manually each time.

The macro is based on a series of automatic steps, requiring the input of the user only when defining the backgrounds of the images (blue and green) (e.g. Fig. S2.1).

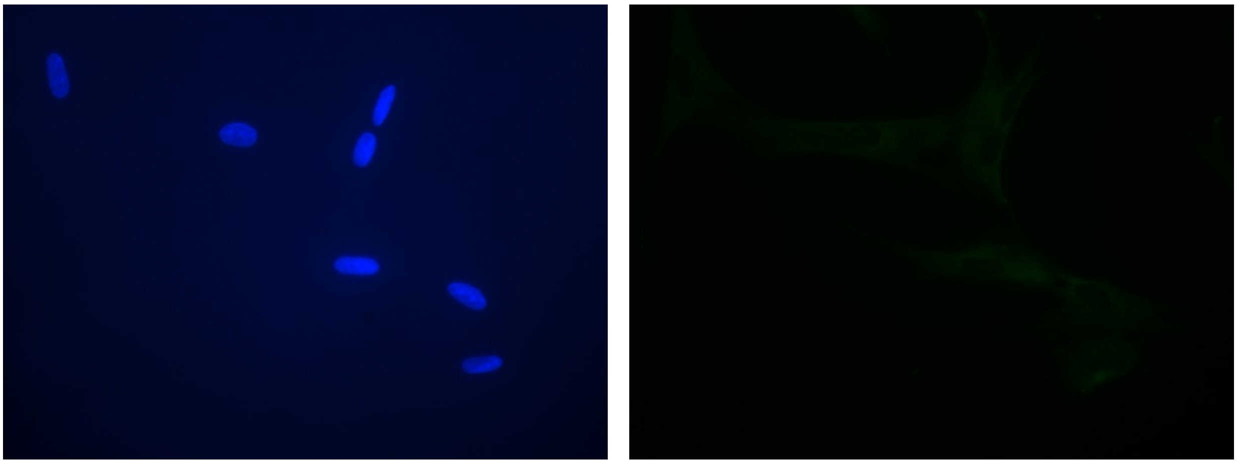

Fig. S2.1 Example of images taken with a 40x magnification. Left: nuclei counterstained with the nuclear dye Hoechst 33342; right: NF-kB-p65 stained cells.

The flow of the analysis is as follows:

- the "Hoechst" image is opened and splitted in the three RGB channels, discarding the red and green and keeping the blue one;
- the user is asked to digitize a proper threshold to be applied to the image, in order to distinguish the nuclei from the background and select their borders as Region Of Interest (ROI) (See Fig. S2.2). During this iterative procedure (ImageJ will provide a preview of the image and requires a confirmation of the selected threshold value before continuing) the user also can modify the circularity and size intervals criteria to discard artifacts (if any);

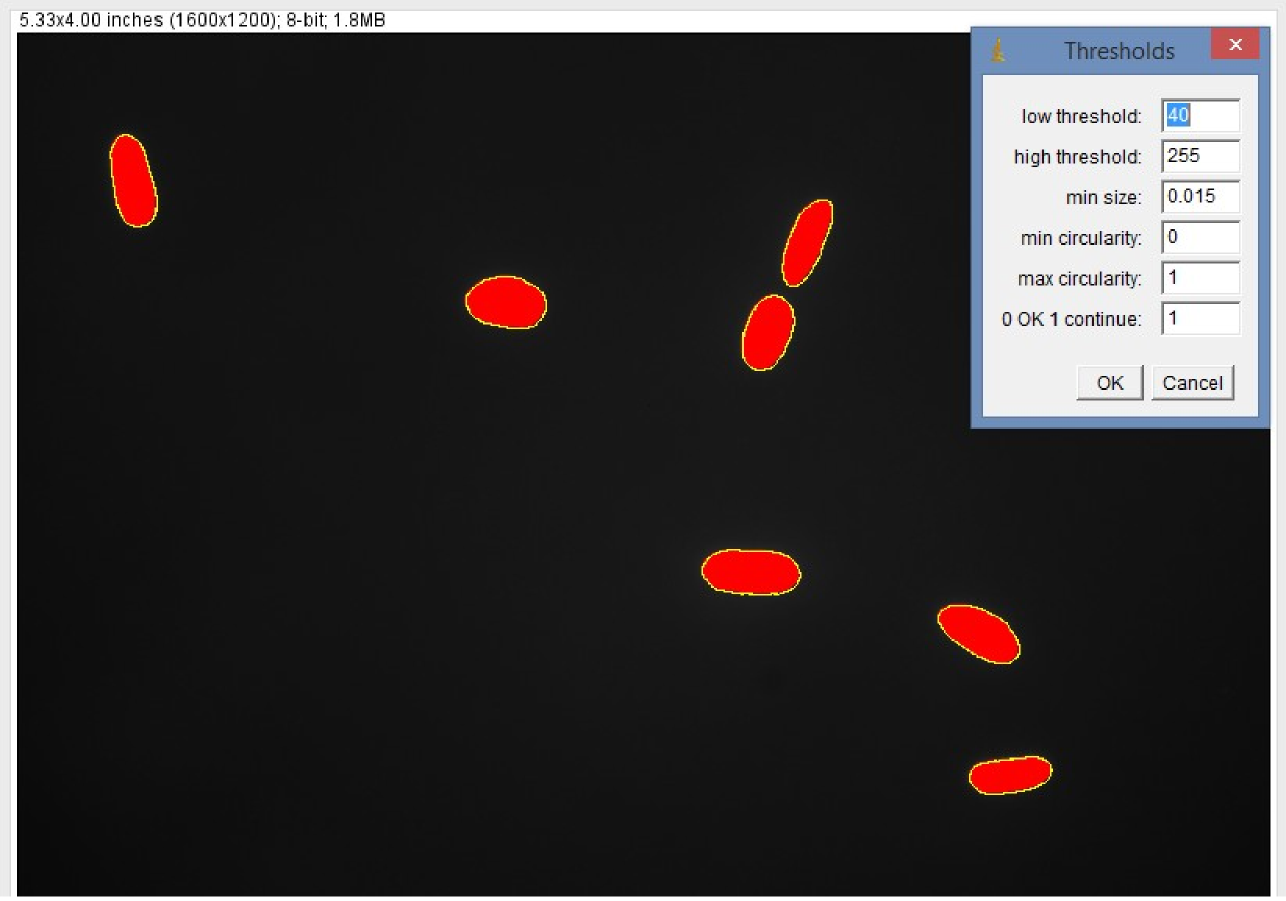

Fig. S2.2 Screenshot of the ImageJ script asking the operator to select the proper threshold for the detection of nuclear margins.

- once the proper background threshold has been selected (confirmation given by the user), ROIs are saved;
- the corresponding "green" image is opened, keeping only the green channel, and saved ROIs are applied to it;
- As shown in Fig. S2.3, the user is asked to select the proper threshold for the image, in order to distinguish the cytoplasm from the background (again with an iterative procedure, during which a preview of the threshold effect is shown at each choice);

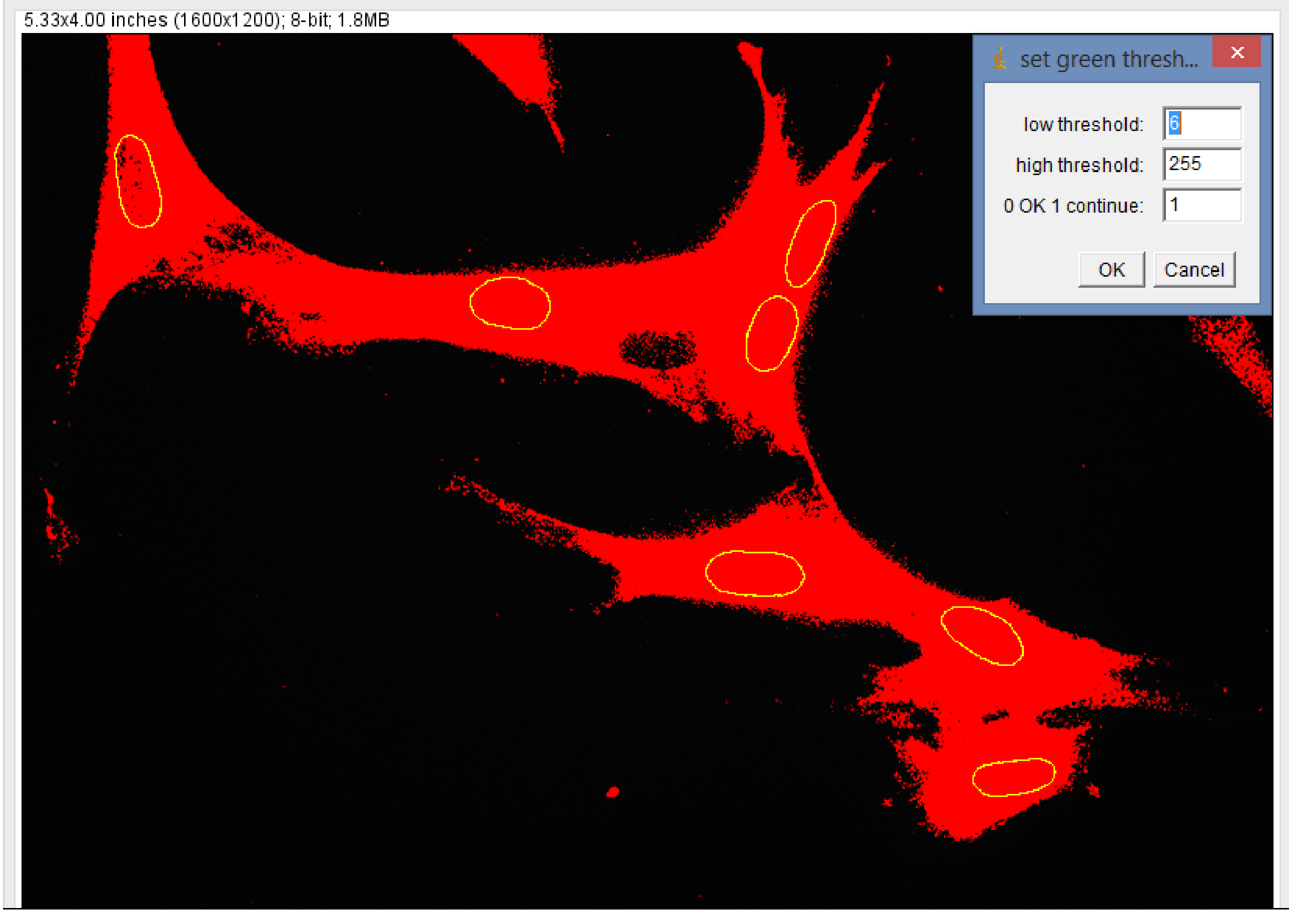

Fig. S2.3 Screenshot of the ImageJ script asking the operator to select the proper threshold for the NF-kB staining.

- once the threshold is confirmed, all the information connected to the analyzed images are recorded. In particular, the total NF-kB intensities inside and outside the ROIs (corresponding, respectively, to the integral fluorescence in the nuclear and cytoplasmic compartments) are stored;

- ROIs are discarded, the following pair of images is read in and made ready to be analyzed through the same steps;

- at the end of the 10 fields (for each slide), an average R ratio is calculated from stored NF-kB intensities in and outside the nuclei, together with the corresponding standard error.

BIBLIOGRAPHY

Baecker, V., & Travo, P. (2006). Cell Image Analyzer—a visual scripting interface for ImageJ and its usage at the microscopy facility Montpellier RIO Imaging. In *Proceedings of the ImageJ User and Developer Conference* (pp. 105–110).
